# Morphine Addiction and Oxidative Stress: The Potential Effects of Thioredoxin-1

**DOI:** 10.3389/fphar.2020.00082

**Published:** 2020-02-21

**Authors:** Xian-Si Zeng, Wen-Shuo Geng, Zhan-Qi Wang, Jin-Jing Jia

**Affiliations:** ^1^ Key Laboratory of Tea Plant Biology of Henan Province, College of Life Sciences, Xinyang Normal University, Xinyang, China; ^2^ Key Laboratory of Vector Biology and Pathogen Control of Zhejiang Province, College of Life Sciences, Huzhou University, Huzhou, China

**Keywords:** morphine addiction, oxidative stress, CPP, thioredoxin-1, geranylgeranylacetone

## Abstract

Long-term administration of morphine for the management of chronic pain will result in tolerance to its analgesic effect and could even cause drug dependence. Numerous studies have demonstrated significant redox alteration in morphine dependence and addiction. Thioredoxin-1 (Trx-1) play important roles in controlling the cellular redox balance. In recent years, several recent studies have demonstrated that Trx-1 may be a promising novel therapeutic target for morphine addiction. In this article, we firstly review the redox alteration in morphine addiction. We also summarize the expression and the protective roles of Trx-1 in morphine dependence. We further highlight the protection of geranylgeranylacetone (GGA), a noncytotoxic pharmacological inducer of Trx-1, in morphine-induced conditioned place preference. In conclusion, Trx-1 may be very promising for clinical therapy of morphine addiction in the future.

## Introduction

Morphine, the most effective opioid analgesic, is clinically used for severe acute and chronic pain. An increasing number of studies have clarified that morphine could display beneficial protection. Morphine at low concentrations promoted cell proliferation and suppressed nicotine-induced cytotoxicity and cell death in PC12 cells (Amini et al., 2019). Low-dose morphine played a neuroprotective role in cellular and animal models of Parkinson's disease through inhibiting oxidative stress and endoplasmic reticulum stress, promoting autophagy activation, and improving mitochondrial function (Wang et al., 2018). Acute administration with morphine alleviated 1-methyl-4-phenyl-1, 2, 3, 6-tetrahydropyridine-induced tremor symptoms in monkeys (Yan et al., 2014). In addition, morphine protected PC12 cells against the cytotoxicity of 1-methyl-4-phenylpyridinium through activating phosphatidylinositol 3-kinase (PI3K)/Akt pathway (Fan et al., 2019). Some groups demonstrated that preconditioning with morphine alleviated cerebral ischemia injury through activating the mTOR pathway (Arabian et al., 2018b) or mitochondrial KATP channels (Arabian et al., 2018a). However, repeated use of morphine will lead to various side-effects, such as antinociceptive tolerance, dependence, and addiction.

## Morphine Addiction

Long-term treatment with morphine for the management of chronic pain will result in tolerance to the analgesic effect of morphine. In order to overcome tolerance, a higher dose of morphine is often required for the maintenance of analgesia, which will result in the development of severe side-effects, including respiratory depression, withdrawal symptoms, and rewarding effects with a high risk of relapse (Eidson and Murphy, 2019). Morphine addiction has become a major public health issue. An increasing number of studies have revealed that several brain regions, such as the ventral tegmental area (VTA), nucleus accumbens (NAc), and hippocampus (Hipp), are involved in morphine addiction (Kim et al., 2016). Although mechanisms underlying morphine-mediated processes remain the subject of much debate, morphine stimulation activates G protein-coupled opioid receptors and then induces significant molecular changes inside the cell, such as an inhibition of adenylate cyclase activity, and activation of potassium channels (Qu et al., 2017; Yang et al., 2019). In addition, other signalling pathways, including mitogen-activated kinases (MAPK), β-arrestin, phospholipase C, protein kinase, PI3K, and extracellular signal-regulated kinase (ERK) pathways, are also involved in morphine activity (Bianchi et al., 2010; Zhang and Pan, 2010; Dai et al., 2018; Shen et al., 2018; de Freitas et al., 2019; Dekan et al., 2019; Listos et al., 2019). Recently, the role of oxidative stress in morphine action has been paid more attention.

### Oxidative Stress in Morphine Addiction

A growing body of evidence has indicated that oxidative stress is involved in the development of addiction with several addictive drugs, including cocaine, methamphetamine, and morphine (Kovacic, 2005; Cai et al., 2016; Jang et al., 2017). Morphine could activate opioid receptors, and its treatment not only promoted the generation of free radicals, including reactive oxygen (ROS) or reactive nitrogen (RNS) species, but also decreased the activities of antioxidants in target cells (Skrabalova et al., 2013). Morphine could induce ROS generation in a time- and concentration-dependent manner in SH-SY5Y cells and excessive ROS subsequently affected morphine addiction through involving µ-opioid receptors (Ma et al., 2015). Systemic morphine use also led to oxidative stress in animals. Oxidative stress levels were increased in the prefrontal cortex and Hipp of morphine-dependent rats (Famitafreshi and Karimian, 2018). Abdel-Zaher and coworkers reported that glutamate levels and lipid peroxide malondialdehyde (MDA) levels were progressively increased in the brain of morphine-treated mice (Abdel-Zaher et al., 2013b). What's more, brain intracellular reduced glutathione (GSH) levels and glutathione peroxidase (GSH-Px) activity were decreased in mice (Abdel-Zaher et al., 2013a). In rats, subcutaneous injection of morphine also significantly increased lipid peroxidation, and decreased the activities of SOD and GSH-Px (Motaghinejad et al., 2015a). GSH levels were depleted in cerebrospinal fluid sampled from cancer patients administrated with morphine intracerebroventricularly, which might render the central nervous system vulnerable to damage from oxidative stress (Goudas et al., 1999). Morphine could alter intracellular levels of GSH-based cellular redox status, subsequently affect S-adenosylmethionine levels (Trivedi and Deth, 2014), and finally induce global DNA methylation changes (Trivedi et al., 2014).

In addition, morphine also affected other oxidative stress-related proteins. Long-term treatment with morphine not only increased the MDA level, but also decreased activities of SOD, glutathione-s-transfrase (GST), and catalase (CAT) in the liver of rats (Samarghandian et al., 2014). Morphine accelerated the disease progression of HIV-infection in macaques due to the deteriorated oxidative stress, including the 50% drop of CAT and SOD (Perez-Casanova et al., 2007). In rat primary neuronal striatal cells, three oxidative stress-related proteins - glyceraldehyde-3-phosphate dehydrogenase (GAPDH), dihydrolipoyl dehydrogenase (DLDH), and aldehyde dehydrogenase (ALDH) - were significantly upregulated after morphine administration (Bodzon-Kulakowska et al., 2009). Proteomic analysis demonstrated that oxidative stress-related proteins, such as peroxiredoxin-2 and Heat shock protein 70 (Hsp70), were significantly decreased in the NAc of morphine-dependent monkeys (Bu et al., 2012) (**Table 1**).

**Table 1:** Morphine-induced oxidative stress in cells, rodents, non-human primates and human.

Species	Morphine treatment	Effects	References
human SH-SY5Y cells	50 μM for 24 hour	↑ROS generation	(Ma et al., 2015)
Rat primary neuronal striatal cells	10 μM for 5 days	↑GAPDH, DLDH and ALDH	(Bodzon-Kulakowska et al., 2009)
Rats	5 mg/kg for 14 days (i.p.)	↑MDA, ↓GSH	(Famitafreshi and Karimian, 2018)
Rats	4 mg/kg for 1^st^ 10 days, 8 mg/kg 2^nd^ 10 days and 12 mg/kg for 3^rd^ 10 days (i.p.)	↑liver MDA and nitric oxide, ↓liver SOD, GST and CAT	(Samarghandian et al., 2014)
Rats	45 mg/kg for 4 weeks (s.c.)	↑lipid peroxidation, ↓SOD and GSH-Px	(Motaghinejad et al., 2015a)
Mice	5 mg/kg twice daily for 7 days (s.c.)	↑MDA and nitric oxide, ↓GSH and GSH-Px	(Abdel-Zaher et al., 2013a; Abdel-Zaher et al., 2013b)
Rhesus monkeys	3 mg/kg (day 1–7), 6 mg/kg (day 8–14), 9 mg/kg (day 15–21), 12 mg/kg (day 22–28), 15 mg/kg (day 29–90) (s.c.)	↓peroxiredoxin-2 and Hsp70	(Bu et al., 2012)
Macaques	5 mg/kg (TID) for 20 weeks (i.m.)	↑MDA, ↓CAT and SOD	(Perez-Casanova et al., 2007)
Cancer patients	0.3 mg (icv.)	↓cerebrospinal fluid GSH	(Goudas et al., 1999)

i.p., intraperitoneally; s.c., subcutaneously; i.m., intramuscularly; icv., intracerebroventricularly.

### Maintaining Redox Balance Inhibits Morphine Action

Given the redox alteration in morphine action, antioxidants may provide a protective role in morphine addiction. SOD is an important intracellular antioxidant. Repeated doses of morphine in mice significantly decreased the activity of the mitochondrial isoform of MnSOD in the dorsal horn of the spinal cord due to the nitration of MnSOD by morphine (Muscoli et al., 2007). SOD-mimetic-agent-injection attenuated the effects of morphine on mitochondrial SOD activity (Motaghinejad et al., 2015b). MnSOD overexpressed by recombinant herpes simplex virus in the periaqueductal gray of morphine-withdrawn rats suppressed the upregulated mitochondrial superoxide and the activation of endoplasmic reticulum stress (Iida et al., 2017).

Numerous studies have demonstrated that exogenous agents providing antioxidant activity could also inhibit the action of morphine. The accepted antioxidant, N-acetyl-cysteine, reversed the down-regulation of antioxidant genes (CAT and CuSOD) in SH-SY5Y cells treated with morphine (Saify et al., 2016). The antidepressant Venlafaxine prevented morphine antinociceptive tolerance at least partly because of its antioxidative properties, including the down-regulation of MDA and inhibition of total thiol and GSH-Px levels in the brains of mice (Mansouri et al., 2018). Fluoxetine, another prescribed antidepressant, also seemed a promising adjuvant to opioid analgesics due to its inhibition of morphine-induced changes in prooxidant-antioxidant balance (Hamdy et al., 2018). Atorvastatin, a lipid-lowering medication, could exhibit protective effects against both tolerance to antinociceptive effects of morphine and withdrawal-induced behaviors *via* normalizing the increased MDA in withdrawn mice (Pajohanfar et al., 2017). The polyphenol curcumin, the most abundant component of traditional Chinese medicine *Curcuma longa*, has antioxidant, anti-apoptotic, anti-inflammatory, immunomodulatory, anticancer, and neuroprotective properties. Curcumin lowered the increased lipid peroxidation and mitochondrial GSSG (oxidized GSH) levels in morphine-treated rats (Motaghinejad et al., 2015a) and attenuated morphine tolerance and dependence by inhibiting the activity of Ca^2+^/calmodulin-dependent protein kinase II α (Hu et al., 2015). These studies suggest that the blocking effects of antioxidants to the action of morphine may provide a promising therapeutic strategy.

## Thioredoxin

The thioredoxin (Trx) system, comprising Trx, thioredoxin reductase (TrxR), and coenzyme NADPH, plays a critical role in maintaining the cellular environment in a reduced state in both prokaryotes and eukaryotes (Holmgren and Lu, 2010). Human Trx is a 12 kDa multifunctional protein with a conserved redox catalytic site (-Cys-Gly-Pro-Cys-) (Bai et al., 2003). The mutual transformation of dithiol and disulfide means Trx plays a vital role in regulating cellular redox balance (Jia et al., 2019). Trx-1, a major isoform located to cytoplasm, can directly scavenge ROS induced by a wide variety of stressors, such as UV irradiation and viral infections. Trx-1 can also inhibit cellular apoptosis (Zeng et al., 2015) because it acts as an endogenous negative regulator of apoptosis signal-regulating kinase 1 (ASK1) in the cytoplasm and inhibits ASK1-dependent apoptotic pathway (Saitoh et al., 1998). It has been reported that Trx-1 expression is enhanced in both chronic and acute stress models and attenuate epinephrine stress-induced DNA damage *via* the negative regulation of β-arrestin-1 (Jia et al., 2014; Jia et al., 2016). Our previous studies have demonstrated that Trx-1 shows a neuroprotective role in central nervous system diseases, including Parkinson's disease and cerebral ischemia (Zeng et al., 2014; Zeng et al., 2018). Interestingly, Trx-1 is involved in the addiction of drugs, including morphine (Luo et al., 2013; Guo et al., 2018).

### The Increased Expression and the Role of Trx-1 Upon Morphine Administration

So far, only a few studies have reported that Trx-1 expression is increased upon morphine administration. Trx-1 was induced through opioid receptors and the activation of PI3K and ERK pathways in morphine-treated SH-SY5Y cells (Luo et al., 2012a). Morphine exposure increased the expression of Trx-1 in dentate gyrus (DG, a brain region involved in memory consolidation), which was reversed by the pretreatment of a corticotropin-releasing factor 1 receptor (CRF1R) antagonist, CP-154,526, with no changes in the paraventricular nucleus (PVN) (Garcia-Carmona et al., 2015). García-Carmona and coworkers found that phosphorylated cAMP-responsive element-binding protein (p-CREB) positive neurons in DG also expressed Trx-1 (Garcia-Carmona et al., 2015), suggesting that Trx-1 could activate CREB and increase the rewarding effects of morphine (**Table 2**). The results are consistent with another study in which Trx-1 ameliorated the learning and memory deficits in a mouse model of Parkinson's disease *via* the restoration of p-CREB in the Hipp (Zhang et al., 2018).

**Table 2:** The effects and molecular mechanisms of Trx-1 and GGA on morphine addiction.

Brain areas	Effects	Mechanisms	References
DG	Morphine-induced increase of Trx-1 enhanced the rewarding effects	Activating CREB	(Garcia-Carmona et al., 2015)
VTA and NAc	Overexpression of Trx-1 inhibited morphine-induced CPP	Upregulating the endogenous concentration of GABA and the expression of GABA_B_ receptor	(Li et al., 2018)
NAc	Inhibiting CPP and attenuating the naloxone-induced withdrawal syndrome	Suppressing the activation of CREB, and the expression of ∆FosB and cyclin-dependent kinase 5	(Luo et al., 2012b)
NAc and hippocampus	Inhibiting morphine reinstatement-induced CPP	Attenuating the activation of NR2B/p-CaMKII/p-ERK/p-CREB pathway	(Guo et al., 2018)

Morphine also markedly increased the expression of Trx-1 in the nucleus accumbens (NAc) of C57BL/6 mice (Luo et al., 2012b). Interestingly, the Trx-1 expression showed a notable elevation in the liver and kidney of morphine-treated mice (Luo et al., 2013). Trx-1 expression was induced by morphine in the ventral tegmental area (VTA) and NAc of mice (Li et al., 2018), two brain regions involved in morphine-induced conditioned place preference (CPP) for both opiates and psychostimulants (Edwards et al., 2017; Zhang et al., 2019). Li et al. further clarified that Trx-1 overexpression in transgenic mice inhibited morphine-induced CPP through upregulating the endogenous concentration of γ-aminobutyric acid (GABA) and the expression of GABA_B_ receptor in the VTA and NAc (Li et al., 2018) (**Table 2**). Considering the critical role of Trx-1 in maintaining the cellular redox state, the increase of Trx-1 expression in morphine-induced CPP might be a compensatory mechanism of stress systems for the maintenance of neuroprotection.

### The Effects of Geranylgeranylacetone on Morphine Treatment

Geranylgeranylacetone (GGA) is a clinical drug, extensively used for ulcer therapy (Ooie et al., 2001). Now GGA has become an accepted pharmacological inducer of Trx-1 (Tanito et al., 2005). Luo et al. demonstrated that pre-treatment with GGA significantly reduced morphine-induced locomotion, inhibited the CPP, and attenuated the naloxone-induced withdrawal syndromes, such as jumping, forepaw tremor, and rearing, through suppressing the activation of CREB, and inhibiting the expressions of ∆FosB and cyclin-dependent kinase 5 in the NAc of C57BL/6 mice (Luo et al., 2012b). Interestingly, the effect of increased Trx-1 by GGA on the activation of CREB in the NAc is contrary to that by CP-154,526 in DG (Garcia-Carmona et al., 2015). In addition, GGA also inhibited reinstatement of morphine-induced CPP through strengthening the expression of Trx-1 and regulating the N-methyl d-aspartate receptor 2B subunit (NR2B)/ERK pathway in the NAc and Hipp, a brain region participating in associative processes such as declarative memory (Guo et al., 2018) (**Table 2**), suggesting that GGA may be a promising therapeutic drug for morphine-induced relapse. These studies suggest that enhancement of Trx-1 expression in the brain by using noncytotoxic pharmacological inducers may provide a novel therapeutic strategy for morphine dependence.

## Conclusion and Expectation

In summary, chronic morphine treatment has been shown to lead to oxidative stress, which plays an important role in the development of morphine tolerance and dependence. An increasing number of studies have clarified that maintaining redox balance through restoration of endogenous antioxidant proteins or treatment with antioxidant agents inhibits the action of morphine. As an antioxidant protein, Trx-1 could effectively inhibit the effects of morphine administration. In this article we reviewed that overexpression of Trx-1 or enhancement of Trx-1 expression by GGA, the noncytotoxic pharmacological inducer of Trx-1, inhibited morphine-induced CPP. At this stage, the studies are extremely few and limited to focusing on the effects of Trx-1 on morphine addiction mainly in rodent models. Their effects on morphine withdrawal and relapse should be investigated in future research. Besides that, nonhuman primate models of morphine addiction should be also developed to accelerate the clinical application of Trx-1 in the future. Trx-1 will provide a novel therapeutic strategy for morphine abuse.

Remarkably, GGA is also the pharmacological inducer of Hsp70, a soluble intracellular chaperone protein (Lennikov et al., 2013). Although GGA was reported to protect mice against morphine-induced hyperlocomotion, rewarding effect, and withdrawal syndromes, as well as morphine-induced hepatic and renal damage (Luo et al., 2012b; Luo et al., 2013), GGA-induced Hsp70 expression in the core of NAc promoted the development of behavioral sensitization, an important behavioral characteristic of drug-addicted animals, providing a biological target for long-lasting adaptations with relevance to morphine addiction (Wang et al., 2014).Recent research has reported that pre-treatment with an Hsp70 transcriptional inducer GGA promoted the development of morphine analgesic tolerance (Qin et al., 2019), suggesting that GGA is not clinically beneficial to the analgesic effect of morphine. Regarding these effects of GGA, further studies are needed to develop much more optimal pharmacological inducers of Trx-1.

## Author Contributions

X-SZ and J-JJ conceptualized the idea for the article. X-SZ and W-SG wrote the final manuscript. X-SZ, Z-QW, and J-JJ revised the manuscript.

## Conflict of Interest

The authors declare that the research was conducted in the absence of any commercial or financial relationships that could be construed as a potential conflict of interest.
